# P066. Migraine with aura in the locker room: 4 case reports

**DOI:** 10.1186/1129-2377-16-S1-A169

**Published:** 2015-09-28

**Authors:** Laura Bernetti, Ilenia Corbelli, Michele Romoli, Chiara Bedetti, Elona Brahimi, Paola Sarchielli, Paolo Calabresi

**Affiliations:** Headache Centre, Neurologic Clinic, “S. M. della Misericordia” Hospital, University of Perugia, Perugia, Italy

We describe four cases of young men 19, 21, 23 and 25 years old with recurrent episodes of migraine with aura occurring shortly after the end of physical activities (football match, swimming, gym training, physical education activities at school), when they were in the locker room.

Since these types of symptoms could mime some important pathologies in approximately 10% of these headaches, it is mandatory, in this kind of patient, to exclude a form of secondary headache [1]. No other subtypes of headache, or head trauma were reported by the patients.

It is well known that physical activity can lead to an aggravation of the intensity of the headache, but the pathophysiological relationship between exertion and aura is unknown and still debated.

There are anecdotal reports of episodes of migraine preceded by head trauma and visual symptoms (with a past history of non-sports-related migraine) [2, 3], migraine prodrome symptoms after unusually strenuous running with no following head pain [4] or recurrent attacks of hemiplegic migraine induced only by exertion [5].

According to the present version of the International Classification of Headache Disorders, (ICHD-III beta), the headache subtype presented by the four patients fulfilled criteria for “migraine with aura” (ICHD-III beta code: 1.2) and for “primary exertional headache” (pulsating headache, lasting from 5 minutes to 48 hours, brought on by and occurring only during or after physical exertion; ICHD-III beta code: 4.2) [6].

To date, in the IHS Classification (ICHD-III beta), there is no mention of sport/exercise-induced migraine with aura episodes as primary headache, and there is the need of a double diagnosis, although there are anecdotal reports of attacks of migraine with aura shortly after sports.

Written informed consent to publish was obtained from the patient(s).
